# Chronic anemia is associated with systemic endothelial dysfunction

**DOI:** 10.3389/fcvm.2023.1099069

**Published:** 2023-05-10

**Authors:** Ramesh Chennupati, Isabella Solga, Patricia Wischmann, Paul Dahlmann, Feyza Gül Celik, Daniela Pacht, Aslıhan Şahin, Vithya Yogathasan, Mohammad Rabiul Hosen, Norbert Gerdes, Malte Kelm, Christian Jung

**Affiliations:** ^1^ Division of Cardiology, Pulmonology and Vascular Medicine, Medical Faculty, Heinrich-Heine University, Düsseldorf, Germany; ^2^ Department of Internal Medicine II, Heart Center Bonn, University Hospital Bonn, Bonn, Germany; ^3^ Cardiovascular Research Institute Düsseldorf (CARID), Medical Faculty, Heinrich-Heine-University, Düsseldorf, Germany

**Keywords:** anemia, endothelial dysfunction, nitric oxide, reactive oxygen species, n-acetyl cysteine, myeloperoxidase

## Abstract

**Background:**

In acute myocardial infarction and heart failure, anemia is associated with adverse clinical outcomes. Endothelial dysfunction (ED) is characterized by attenuated nitric oxide (NO)-mediated relaxation responses which is poorly studied in chronic anemia (CA). We hypothesized that CA is associated with ED due to increased oxidative stress in the endothelium.

**Methods:**

CA was induced by repeated blood withdrawal in male C57BL/6J mice. Flow-Mediated Dilation (FMD) responses were assessed in CA mice using ultrasound-guided femoral transient ischemia model. Tissue organ bath was used to assess vascular responsiveness of aortic rings from CA mice, and in aortic rings incubated with red blood cells (RBCs) from anemic patients. In the aortic rings from anemic mice, the role of arginases was assessed using either an arginase inhibitor (Nor-NOHA) or genetic ablation of arginase 1 in the endothelium. Inflammatory changes in plasma of CA mice were examined by ELISA. Expression of endothelial NO synthase (eNOS), inducible NO synthase (iNOS), myeloperoxidase (MPO), 3-Nitrotyrosine levels, and 4-Hydroxynonenal (4-HNE) were assessed either by Western blotting or immunohistochemistry. The role of reactive oxygen species (ROS) in ED was assessed in the anemic mice either supplemented with N-Acetyl cysteine (NAC) or by *in vitro* pharmacological inhibition of MPO.

**Results:**

The FMD responses were diminished with a correlation to the duration of anemia. Aortic rings from CA mice showed reduced NO-dependent relaxation compared to non-anemic mice. RBCs from anemic patients attenuated NO-dependent relaxation responses in murine aortic rings compared to non-anemic controls. CA results in increased plasma VCAM-1, ICAM-1 levels, and an increased iNOS expression in aortic vascular smooth muscle cells. Arginases inhibition or arginase1 deletion did not improve ED in anemic mice. Increased expression of MPO and 4-HNE observed in endothelial cells of aortic sections from CA mice. NAC supplementation or inhibition of MPO improved relaxation responses in CA mice.

**Conclusion:**

Chronic anemia is associated with progressive endothelial dysfunction evidenced by activation of the endothelium mediated by systemic inflammation, increased iNOS activity, and ROS production in the arterial wall. ROS scavenger (NAC) supplementation or MPO inhibition are potential therapeutic options to reverse the devastating endothelial dysfunction in chronic anemia.

## Introduction

Endothelial dysfunction (ED) is central to a plethora of cardiovascular diseases (CVD). ED is defined by the inability to maintain vascular homeostasis due to decreased nitric oxide (NO) production (1, 2). NO is a major vasodilator and anti-inflammatory signalling molecule, which plays an important role in vascular tone and blood pressure regulation. Decreased NO bioavailability disrupts the non-thrombogenic intimal surface, which promotes adhesion and aggregation of platelets and monocytes to the activated endothelial surface (3). The bioavailability of NO mainly depends on the availability of eNOS' substrate, arginine, and coupling state of eNOS. Arginases are catabolizing enzymes of arginine, which share the common substrate with eNOS, and are known to limit NO bioavailability (4, 5). Both isoforms of arginase (1 and 2) are expressed in endothelial cells of mice and humans (6). Endothelial arginase 1 is known to contribute to ED in coronary arteries of diabetic patients (7), and animal models of hypertension, ischemia/reperfusion, and aging (8, 9). In addition, endothelial arginase 2 upregulation and associated NO dysregulation have been reported in diabetes, aging (10), and hyperlipidemia (11). Inhibition of arginases led to increased NO-bioavailability and consequently improved the endothelium-dependent relaxation responses in different disease states (8).

The bioavailability of NO depends on the coupling state of eNOS, which relies on several factors such as oxidative depletion of tetrahydrobiopterin (BH4), S-glutathionylation, reactive oxygen, and nitrogen species (12). Concomitantly, uncoupled eNOS in the NO generation system leads to the production of superoxide anions (O_2_^−^) rather than NO (13). Thus, superoxide anions are not only associated with reduced NO production but also enhance pre-existing oxidative stress. Additionally, peroxynitrite is known to cause depletion of cofactor BH_4,_ which leads to uncoupling of eNOS and thus, reduced NO-bioavailability (14). Recent studies also showed that myeloperoxidase (MPO), which is upregulated in endothelial cells during inflammation, limits NO-bioavailability by producing reactive oxidants such as hypochlorous acid (15).

Anemia is frequently observed in patients with CVD such as acute and chronic coronary syndromes as well as heart failure. Approximately 30 to 40% of patients with acute myocardial infarction (AMI) have evident anemia upon admission (16), or during hospitalization (17). It is well-studied that anemia alone or in combination with other morbid conditions leads to poor prognosis in AMI (18). These studies have concluded that dysfunctional RBCs in anemia influence the severity of CVD. We recently showed that acute blood-loss anemia transiently increases FMD due to an acute increase in shear stress and cardiac output. Simultaneously acute anemia induces RBC dysfunction by reducing NO bioavailability, increasing ROS formation, reducing membrane integrity, and increasing NO scavenging by free plasma hemoglobin (19). However, it is unclear whether the complications related to chronic anemia are restricted to RBC dysfunction or also associated with systemic alteration in endothelial cells that may result in ED.

In this study, we evaluated the systemic ED in a newly established murine blood-loss chronic anemia model. Additionally, we also investigated whether the RBCs from anemic patients affect the endothelial function using co-incubation with aortic rings followed by endothelial function analysis. To our knowledge, this is the first study to evaluate systemic ED in a blood-loss anemia murine model.

## Materials and methods

### Animals

All procedures were approved and performed in accordance with the guidelines of LANUV (Landesamt für Natur, Umwelt- und Verbraucherschutz Nordrhein-Westfalen, Germany). Mice were treated by following the European Convention for the protection of Vertebrate Animals used for Experimental and other Scientific Purposes (Council of Europe Treaty Series No. 123). The approved permits for the animal experiments are 84-02.04. 2020.A073 and 84-02.04.2018.A234. The C57Bl/6J (wild type, WT) mice were obtained from Janvier Labs (Saint-Berthevin Cedex, France). Mice with endothelial ablation of arginase 1 (ARG1) were generated by crossing Arg1^fl/fl^ (C57BL/6-Arg1^tm1Pmu^/J, Jackson laboratory) mice with tamoxifen-inducible Cdh5-creERT2 [Tg(Cdh5-cre/ERT2)1Rha, MGI:3848982] mice which were kindly provided by Prof Dr E. Lammert (Heinrich-Heine-University of Düsseldorf). To induce the recombination, the mice were injected with 50 mg/Kg body weight tamoxifen (Sigma) for 5 consecutive days*.* The resulting mice that lack ARG1 in their endothelium Arg1^fl/fl^/Cdh5-cre/ERT2-pos are named as EC-Arg1-KO in the manuscript. The respective Cre-negative littermates were used as controls. All mice were housed in standard cages (constant room temperature and humidity, 12hr light/dark cycles) and had free access to standard pelleted chow and tap water.

### Anemia induction

To induce anemia, WT, EC-Arg1-KO and respective control mice were divided into two groups, a sham group without anemia and a group with induced chronic anemia. To induce chronic anemia, a repetitive mild blood withdrawal (by <20 g/L changes in Hb) from the facial vein was performed every third day for a time period of six weeks. At the end of the 6th week mice which showed hemoglobin levels < 10 g/L, are considered for experiments. The hematological parameters after anemia induction are represented in Supplementary Figure S1.

**Supplementation of NAC:** Male (WT) mice, which were 10–11 week-old, were induced with chronic anemia. After two weeks of anemia induction, anemic and respective sham mice were given 1% (w/v) NAC (Sigma) through drinking water for 4 weeks. After 6 weeks of anemia, mice were sacrificed and tissues were collected.

### Flow-mediated dilatation assessment

The 10–11 week-old C57BL/6J male mice were used for the assessment of flow-mediated dilation (FMD) responses. The measurements were performed before, and after 2-, 4-, 6, -weeks of anemia induction. FMD responses were assessed using the Vevo 2,100 high-resolution ultrasound scanner using a 30–70 MHz linear transducer (Visual Sonics Inc., Toronto, Canada) as described previously (20). Mice were kept under 1.5%–2% isoflurane anesthesia, the transducer was placed using a stereotactic holder and adjusted manually to visualise the external iliac artery. A vascular occluder (8 mm diameter, Harvard Apparatus, Boston, MA, USA) was placed around the lower limb (20). Baseline images of the vessel were first recorded, the cuff was inflated to 200 mmHg, and pressure was kept constant for 5 min (Druckkalibriergerät KAL 84, Halstrup Walcher, Kirchzarten, Germany); then the cuff was released to assess FMD. The upstream diameter of the vessel was determined every 30 s both during inflation and deflation of the cuff. Changes in vessel diameter were quantified as the percentage of baseline (%) = [diameter (max)/diameter (baseline)] × 100.

### *In vitro* studies with isolated aortic rings

### Solutions and drugs

Krebs-Ringer bicarbonate-buffered salt solution (KRB) contained (in mM): 118.5 NaCl, 4.7 KCl, 2.5 CaCl_2_, 1.2 MgSO_4_, 1.2 KH_2_PO_4_, 25.0 NaHCO_3_ and 5.5 glucose. The KRB solution was continuously aerated with 95% O_2_/5% CO_2_ and maintained at 37°C. Indomethacin (INDO; Sigma Aldrich) was dissolved in ethanol. Acetylcholine (ACH), phenylephrine (PHE), NG-nitro-arginine methyl ester (L-NAME) and sodium nitroprusside (SNP; all Sigma Aldrich) were dissolved in KRB solution. Myeloperoxidase inhibitor (AZD5904) was purchased from PromoCell and dissolved in dimethyl sulfoxide (DMSO).

### Organ chamber experiments

Animals were euthanized under deep isoflurane anesthesia (4.5%). The thoracic aorta was dissected free from perivascular adipose tissue, and 2 mm size aortic rings were mounted in an organ bath equipped with force transducers (Hugo Sachs Electronic, HARVARD apparatus GmbH, March-Hugstetten Germany) or wire-myograph system (Danish Myotechnology, Aarhus, Denmark). Arterial segments were distended stepwise to 1 gr force or 9.8 mN. All aortic segments were allowed 40 min incubation prior to experiments and stimulated with depolarizing 60 mM KCl as described previously (21). Prior to experiment, endothelial integrity was checked by assessing relaxation responses to ACH (10 µM) in PHE (10 µM) pre-contracted arteries. The aortic segments were pre-contracted and segments which showed less than 80% relaxation responses were excluded from experiments.

#### Contributions of NO, cyclo­oxygenase products to endothelium-dependent relaxation

Initially, a concentration-response curve (CRC) for PHE (0.001–10 μM) was constructed. During the contraction induced by 10 µM PHE, an ACH CRC (1 nM–10 μM) was generated. Next, experiments were repeated in the presence of the cyclooxygenase inhibitor INDO (10 µM) and in the presence of both INDO and the NOS inhibitor L-NAME (100 µM) to assess the contribution of NO to arterial relaxation.

#### Sensitivity of vascular smooth muscle to NO

During contraction of the aortic rings with PHE (10 µM) in the presence of INDO (10 µM) and L-NAME (100 µM), the relaxing effects of the NO donor SNP (10 nM–10 µM) were recorded.

#### Co-incubation studies with human RBCs

The protocol is based on previous studies (22) with adjustments for murine aortic rings. Briefly, thoracic aortas from 10 to 11 week-old mice were dissected and placed in cold KRB buffer. Anemic and non-anemic patient blood was collected in heparin-coated tubes, and 40% hematocrit (Hct) was prepared with KRB buffer. Aortic segments were transferred to the 40% Hct containing KRB buffer and placed in an incubator for 6 h at 37°C. We previously (unpublished data) assessed the best suitable time for co-incubation experiments based on the viability and maximum responses to agonists (PHE, ACH) of aortic rings. After 6 h of incubation, aortic segments were mounted in the wire-myograph system and endothelial-dependent relaxation responses were assessed. Approval numbers for patient samples collection are 5481R, 2018–14, and 2018–47 issued by clinical ethics committee, Universitätsklinikum Düsseldorf, Germany. All patients were given their written consent according to Helsinki ethical principles.

### Western blot

The thoracic aortas were thawed and homogenized using a mixer mill (MM400 Retsch Haan, Germany) and a brief sonification in a radioimmunoprecipitation assay (RIPA) buffer. For further processing, the protein concentration was determined with the DC™ Protein Assay Kit (Bio-Rad, Feldkirchen, Germany). The samples were denatured and loaded on a 4%–12% Bis-Tris gradient gel QPAGE™ (Smobio, Hsinchu City, Taiwan). Proteins were then transferred onto nitrocellulose membranes and a total protein staining was performed by using Revert Total Protein Stains (LI-COR Biosciences GmbH, Bad Homburg, Germany). The unspecific sites were blocked using intercept TBS Protein-Free Blocking Buffer (LI-COR) for 60 min at RT. Next, the membrane was incubated with either iNOS polyclonal antibody (1:1,000 in blocking buffer; ThermoFisher Scientific, Schwerte, Germany) or 3-Nitrotyrosine monoclonal antibody (1:1,000 in blocking buffer; Santa Cruz Biotechnology, Inc., Heidelberg, Germany) for overnight at 4°C. Afterwards, the membranes were incubated with corresponding secondary antibodies (IRDye® 700/800CW secondary antibodies; LI-COR) for 1 h at room temperature. The membranes were visualized with the Odyssey® Fc Imaging System (LI-COR).

### Immunohistochemistry

Thoracic aortas from anemic and respective sham mice were collected, fixed in formaldehyde (4%) for 2 h, and treated with sucrose (30%) overnight. Next, the aortic segments were embedded in Tissue-Tek O.C.T and frozen until further use. Tissue sections (4 µm) were incubated overnight at 4°C with rat anti-mouse CD31 [1:100 in blocking solution (2.5% BSA); BD Biosciences, Heidelberg, Germany] and either with mouse anti-eNOS/NOS Type III (1:100 in 2.5% BSA; BD Biosciences), mouse anti-iNOS (1:100 in 2,5% BSA; Abcam), rabbit anti-4HNE (1:200 in 2,5% BSA; Abcam) or rabbit anti-myeloperoxidase (1:200 in 2,5% BSA; antibody.com) antibodies. Followed by incubating with respective anti-Mouse Alexa Fluor 555 (1:1,000 in 2.5% BSA; Thermo Fisher), goat anti-rat Alexa Fluor 488 (1:1,000 in 2.5% BSA; ThermoFisher) or anti-Rabbit Alexa Fluor 555 (1:1,000 in 2.5% BSA; ThermoFisher) secondary antibodies. Additionally, autofluorescence was quenched using Vector® TrueVIEW® Autofluorescence Quenching Kit (BIOZOL Vectorlabs, Eching, Germany). The stained tissue sections were mounted with the ProLong™ Diamond Antifade Mountant mounting medium (ThermoFisher) and imaged with a Leica DM6 M microscope.

### ELISA

The blood was collected from different experimental groups, plasma was prepared and snap-frozen. Plasma samples were stored at −80°C until further use. Mouse ICAM-1/CD54, VCAM-1/CD106, ELISA kits were purchased from R&D Systems (Bio-Techne GmbH, Wiesbaden Germany). Additionally, mouse haptoglobin, free hemoglobin, and transferrin plasma levels were assessed by using ELISA kits from Abcam. Mouse soluble transferrin receptor, ferritin and erythropoietin ELISA kits were obtained from MyBioSource (BIOZOL, Eching, Germany). All assays were performed according to the manufacturer's instructions. The plasma inflammatory markers were analyzed using a multiplex system (Bioplex, BioLegend).

### qRT-PCR

After 6 weeks of anemia induction, the thoracic aorta was isolated from anemic and respective sham mice, and snap-frozen until further use. The aortas were thawed and homogenized in a lysis buffer using a mixer mill (MM400 Retsch). RNA was isolated from tissue lysates using RNeasy Mini Kit (Qiagen, Hilden, Germany) following the manufacturer's instructions. Reverse transcription was performed following the manufacturer's instructions using Superscript™ IV VILO™ Master Mix (Invitrogen). The real-time PCR was performed using the QuantStudio-7 Flex (ThermoFisher). The TaqMan probes (ThermoFisher) used for real-time PCR were glyceraldehyde 3-phosphate dehydrogenase (GAPDH) (Mm03302249_g1), MPO (Mm00447886_m1) and iNOS (Mm00440502_m1,). Relative expression levels were obtained by normalization with GAPDH.

### Statistical analysis

All concentration response curves (CRCs) for contractile stimuli are expressed as absolute values. Relaxing responses were expressed as a percentage reduction of the level of contraction. Individual CRCs were fitted to a non-linear sigmoid regression curve (Graphpad Prism 8.0). Data normality was checked with D'Agostino Person test. The normally distributed data for multiple groups were analysed with two-way analysis of variance (ANOVA) with Bonferroni's post-hoc test and significance was calculated for all the concentration–response curves. Comparisons between two groups were performed with unpaired *t*-test or Mann–Whitney test depending on the normality. Sensitivity (pEC_50_), and maximal effect (E_max_) are shown as means ± SEM.

## Results

### Chronic anemia is associated with progressive endothelial dysfunction

**Figure 1:**
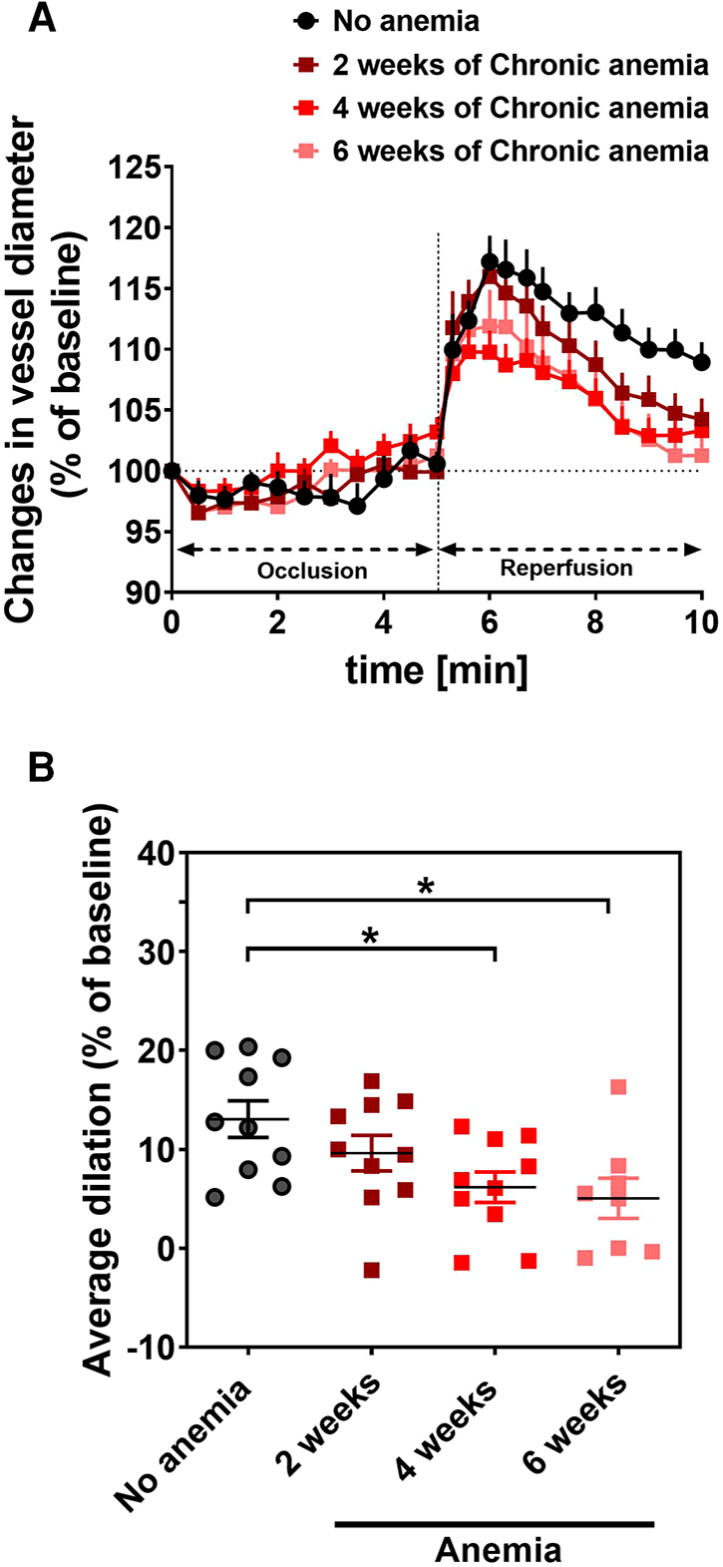
Chronic anemia is associated with altered flow-mediated dilation (FMD) responses. (**A**) Changes in vessel diameter (% ratio of baseline diameter) before (black circles), 2 (dark red squares), 4 (red squares), and 6 weeks (light red squares) after anemia induction. The occlusion and releasing phase of the cuff (reperfusion) are indicated in the panel. (**B**) Average FMD in the reperfusion phase (6–10 min) was normalized to baseline. Values are shown as the means ± SEM (*n* = 10 per group).*, *p* ≤ 0.05. One-Way ANOVA with Sidak's post-hoc test was used to compare average FMD between baseline and respective week.

**Figure 2:**
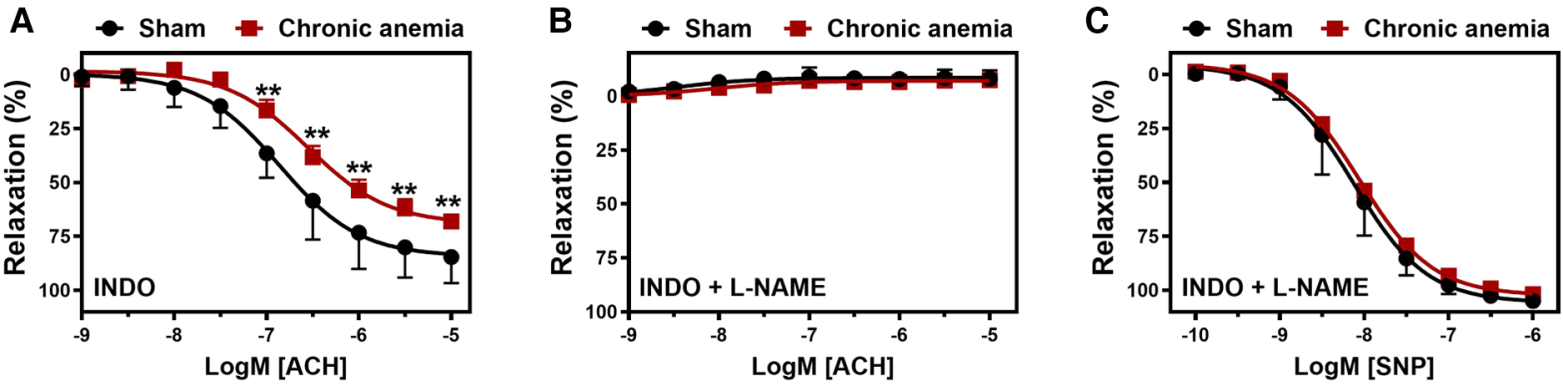
Chronic anemia is associated with reduced endothelial-dependent relaxation responses. Aortic rings were isolated from sham (black circles) and chronic anemic (dark red squares) mice. Aortic segments were pre-contracted with phenylephrine (10 µM) and relaxation responses to acetylcholine (ACH, 1 nM–10 µM) were measured using organ bath. (**A**) relaxation in the presence of indomethacin (10 µM, COX inhibitor). (**B**) relaxation in the presence of indomethacin and L-NAME (100 µM, NOS inhibitor). (**C**) Relaxation responses to sodium nitroprusside (SNP, 10 nM-10 µM) in the presence of indomethacin and L-NAME. All values are mean values ± SEM (*n* = 8–10 per group). **, *p* ≤ 0.01. CRCs were analysed by Two-Way ANOVA and Bonferroni ‘s post-hoc test to compare sham and chronic anemic groups.

To assess the effect of CA on endothelial function *in vivo*, we determined flow-mediated dilation (FMD) responses before induction of anemia (basal) and after 2-, 4-, 6- weeks of anemia. The average FMD responses significantly abrogated after 4- and 6-weeks (6 ± 1% and 5 ± 2%, respectively) of anemia induction compared to before induction of anemia (13 ± 2%) (Figures 1A,B) indicating the progressive ED in CA. Furthermore, we assessed the endothelium-dependent and independent relaxation responses in the isolated aortic rings using an organ bath. The CA mice showed a reduced relaxation (E_max_ 68 ± 4%) response to acetylcholine (ACH) compared to sham mice (E_max_ 84 ± 4%) (Figure 2A; Table 1). Furthermore, in the presence of NOS inhibitor (L-NAME, 100 µM), relaxation responses were completely inhibited in both anemic and sham groups (Figure 2B; Table 1), which shows that the relaxation responses are entirely mediated by NO. In addition, the relaxation responses to exogenous NO donor [Sodium nitroprusside (SNP)] are similar in CA mice compared to the sham group suggesting that smooth muscle sensitivity to NO is unaltered in CA mice compared to sham mice (Figure 2C; Table 1). Taken together, CA mice showed reduced endothelium-dependent relaxation responses. These results demonstrate the altered endothelial function in CA.

**Table 1:** Effect of anemia on endothelial-dependent and -independent relaxation responses.

	pEC50	E_max_ (%)	*n*
**ACH-induced relaxation**			
**INDO**			
Sham	6.86 ± 0.08	84 ± 4	10
Anemia	6.55 ± 0.09^†^	68 ± 4^††^	8
**SNP-induced relaxation**			
Control	8.10 ± 0.04	104 ± 1	10
Anemia	8.00 ± 0.03	102 ± 1	8
**Arginase inhibition**			
**ACH-induced relaxation**			
**INDO**			
Sham	6.86 ± 0.08	85 ± 3	10
Anemia	6.59 ± 0.08	68 ± 5	6
Anemia + Nor-NOHA	6.51 ± 0.07^†^	53 ± 3^††^	6
**Arginase deletion**			
**ACH-induced relaxation**			
**INDO**			
Control + Anemia	6.91 ± 0.11	77 ± 4	5
EC-Arg1-KO + Anemia	6.72 ± 0.11	74 ± 7	5
**SNP-induced relaxtion**			
Control + Anemia	7.76 ± 0.03	107 ± 2	5
EC-Arg1-KO + Anemia	7.68 ± 0.04	109 ± 3	5
**NAC Supplementation**			
**ACH-induced relaxation**			
**INDO**			
Sham + NAC	6.55 ± 0.09	68 ± 4	8
Anemia + NAC	6.76 ± 0.06	81 ± 3†	7
**SNP-induced relaxation**			
Control + NAC	8.06 ± 0.03	102 ± 1	8
Anemia + NAC	7.94 ± 0.04	104 ± 2	7
**MPO inhibition**			
**ACH-induced relaxation**			
**INDO**			
Anemia	6.65 ± 0.11	58 ± 4	6
Anemia	7.04 ± 0.13	79 ± 6	7
**SNP-induced relaxation**			
Anemia	7.57 ± 0.09	100 ± 2	6
Anemia	7.54 ± 0.03	101 ± 3	7

Table 1. The maximal relaxation response (E_max_) are expressed as % reduction of the maximal contractile response to 10 µM PHE. In all experiments, aortic rings did not show any relaxation in the presence of L-NAME so the data is excluded from table. ACH, acetyl choline; INDO, indomethacin; SNP, sodium nitroprusside; MPO, myeloperoxidase; NAC, N-acetyl cysteine. All values are shown as mean ± SEM.

^†^ *P* < 0.05 compared to arteries of sham mice under the same condition. n/a. not applicable.

^††^ *P* < 0.01 compared to arteries of sham mice under the same condition. n/a. not applicable.

### Arginases do not contribute to the altered NO release in chronic anemia

**Figure 3:**
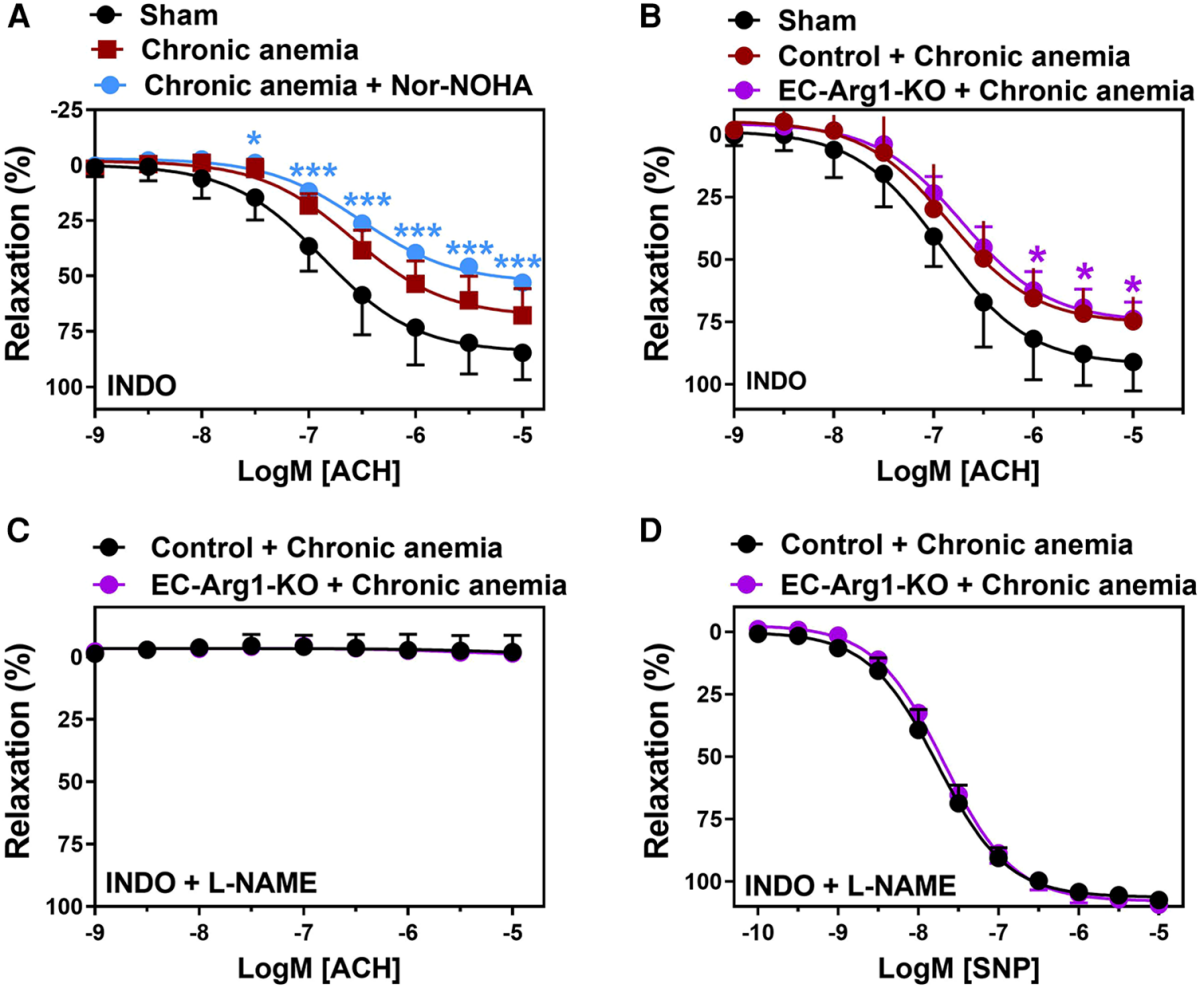
Inhibition of arginases or arginase 1 ablation does not improve endothelium- dependent relaxation responses in anemic mice. (**A**) Aortic rings were isolated from sham (black circles) and chronic anemic mice and incubated in the presence (blue circles) or absence (red squares) of Nor-NOHA (arginase inhibitor, 1 µM). Relaxation responses in the presence of indomethacin (10 µM, COX inhibitor) were measured in phenylephrine (10 µM) pre-contracted arteries. (**B–D**) Aortic rings were isolated from chronic anemic control (dark red circles) and EC-Arg1-KO mice (purple circles). All aortic segments were pre-contracted with phenylephrine (10 µM) and relaxation responses to acetylcholine (1 nM–10 µM) were measured using organ bath. (**B**) Relaxation in the presence of indomethacin (10 µM). (**C**) Relaxation in the presence of indomethacin and L-NAME (100 µM, NOS inhibitor). (**D**) Relaxation responses to sodium nitroprusside (SNP, 10 nM-10 µM) in the presence of indomethacin and L-NAME. All values are mean values ± SEM (*n* = 6–10 per group). *, *p* ≤ 0.05; ***, *p* ≤ 0.001. CRCs were analysed by Two-Way ANOVA and Bonferroni's post-hoc test to compare anemic Nor-NOHA treated or anemic EC-Arg1-KO with sham mice.

Arginase 1 activity is known to be enhanced in various pathological states and is also known to limit NO bioavailability (4, 23). We assessed the relaxation responses after arginase inhibition in anemic mice. The aortic segments from anemic mice treated with Nor-NOHA (non-specific inhibitor of Arginase 1 & 2) did not show any improvement in the relaxation responses (E_max_ 53 ± 3%) compared to untreated (E_max_ 68 ± 5%) aortic segments (Figure 3A; Table 1). In addition, endothelial-dependent relaxation responses are not improved in anemic EC-Arg1-KO (E_max_ 74 ± 6%) and respective anemic control mice (E_max_ 77 ± 4%) compared to the sham mice (E_max_ 91 ± 5%) (Figure 3B). These relaxation responses are mediated by NO (Figure 3C; Table 1). The endothelium-independent relaxation to SNP was comparable in both groups (Figure 3D; Table 1). Taken together, these results exclude the potential role of arginases in ED associated with anemia.

### Chronic anemia is associated with enhanced iNOS expression in vascular smooth muscle cells

**Figure 4:**
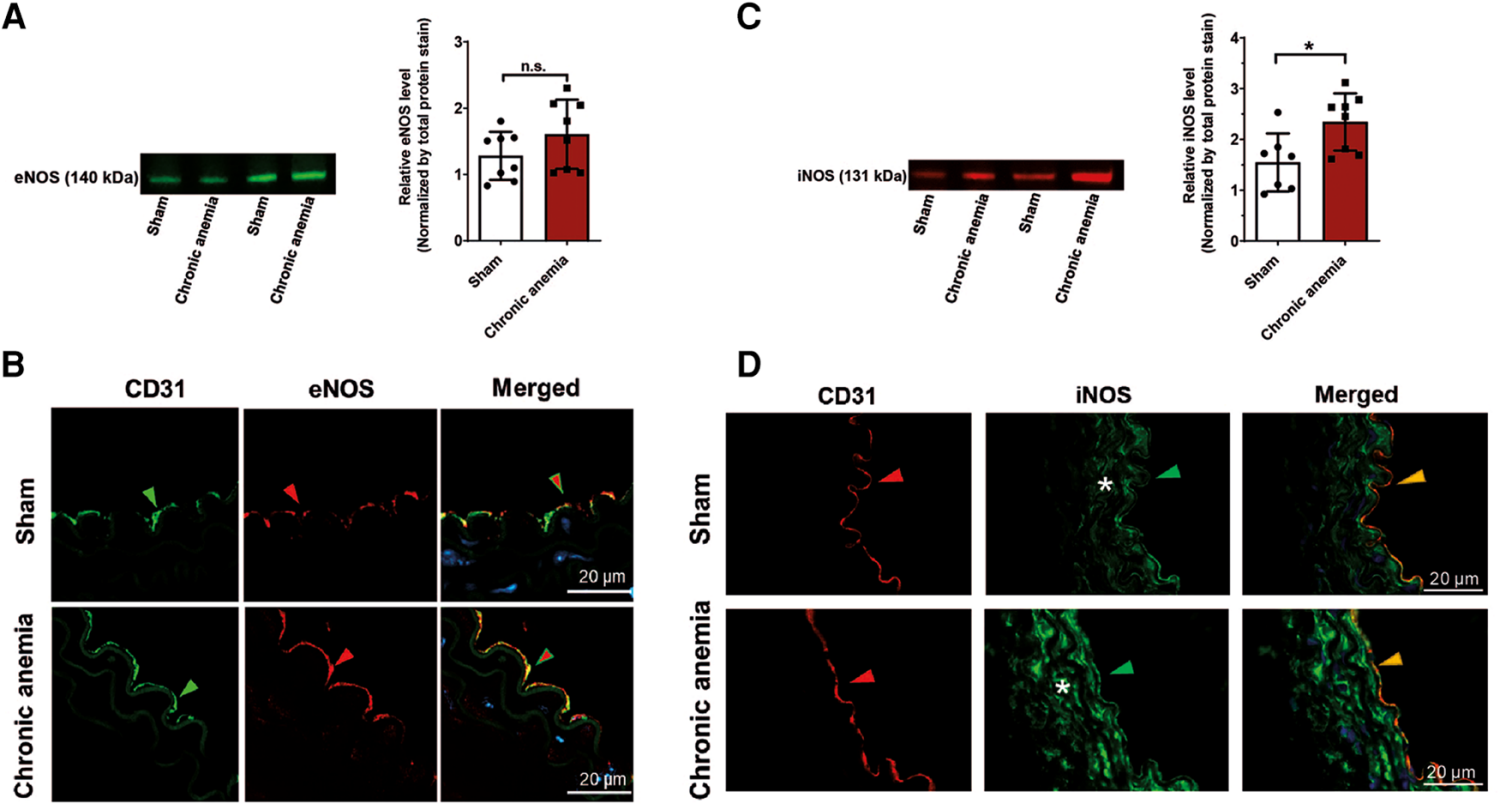
Expression of iNOS is increased in chronic anemic mice. (**A,C**) Representative Western blot images showing protein expression levels and quantification of eNOS (**A**) and iNOS (**C**) in the thoracic aortas isolated from sham and chronic anemic mice. Values are shown as means ± SEM (*n* = 7–8 per group). *, *p* ≤ 0.05. Unpaired *t*-test (**A**) or Mann–Whitney test (**C**) was used to compare between two groups. (**B,D**) Representative immunohistochemistry images showing the expression of CD31, eNOS, or iNOS. DAPI (blue) staining is used to detect the nucleus. Arrowheads are in the luminal side pointing towards the respective staining. Asterisk represents the smooth muscle area. Results are representative pictures of 3 (sham) and 3 (chronic anemic) independent experiments.

To evaluate whether the reduced relaxation responses in CA are also associated with decreased eNOS abundance, we analyzed the protein expression of eNOS in aortic lysates from anemic and respective sham mice. Interestingly, the eNOS levels are similar in CA mice and sham mice (Figure 4A, Supplementary Figure S5). In addition, immunohistochemical analyses also demonstrated similar patterns of eNOS expression in CA and sham mice (Figure 4B). We further assessed the levels of iNOS, a signature molecule for vascular inflammation. The aortic lysates from CA mice showed enhanced iNOS expression compared to sham mice (Figure 4C, Supplementary Figure S5). The immunohistochemistry analysis showed that iNOS expression is increased in vascular smooth muscle cells of CA mice compared to sham mice (Figure 4D).

### Chronic anemia is associated with enhanced inflammation and oxidative stress

**Figure 5:**
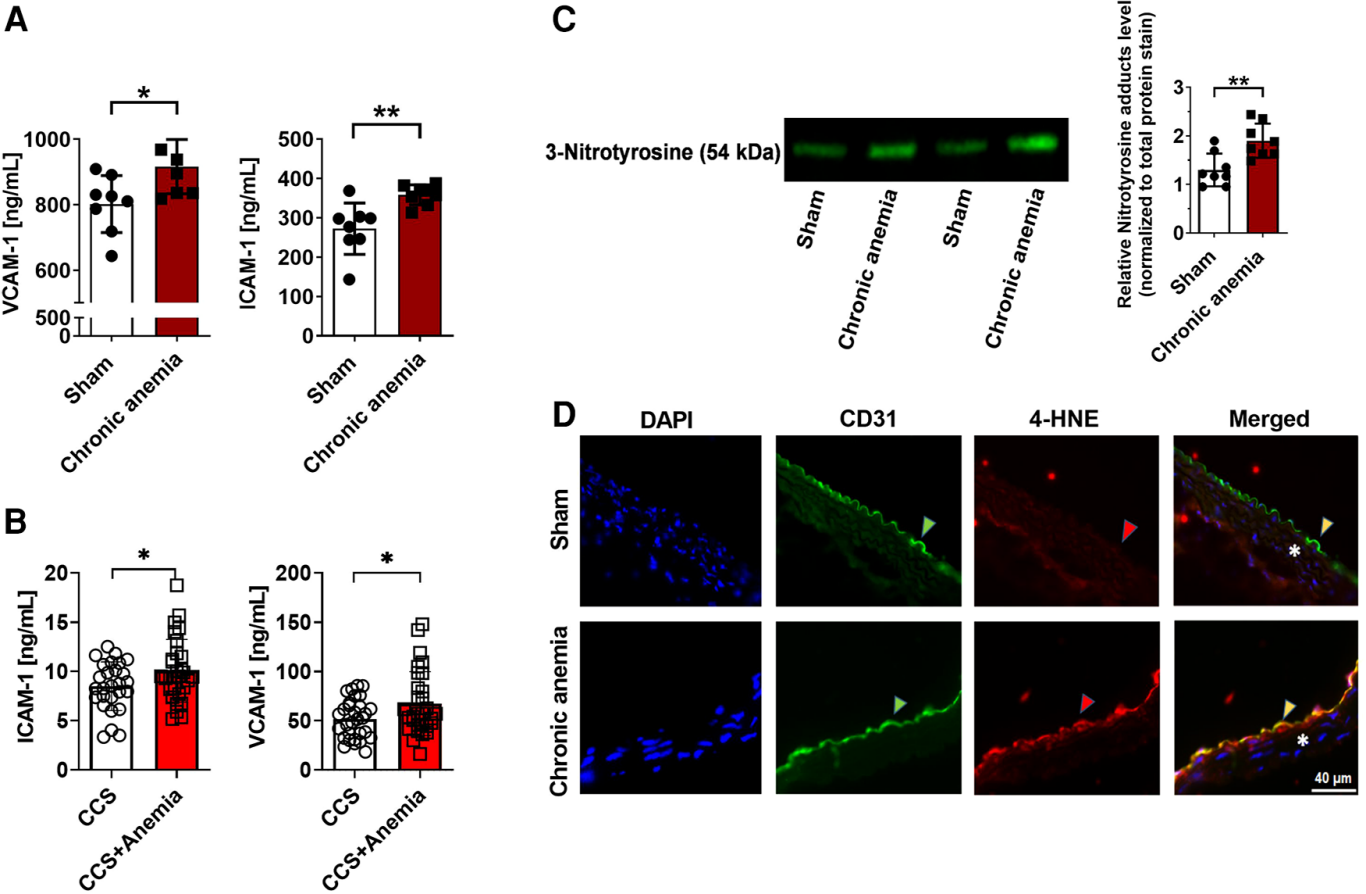
Chronic anemia is associated with increased inflammation and ROS formation. (**A**) Plasma VCAM-1 and ICAM-1 levels in sham (white bars), and chronic (dark red bar) anemic mice. (**B**) Plasma VCAM-1 and ICAM-1 levels in CCS patients without anemia (white bars) and with anemia (red bar). (**C**) Expression of nitrotyrosine protein adducts in the aortic lysates from sham (white bar), and anemic mice (red bar). Values are shown as means ± SEM. *, *p* ≤ 0.05; **, *p* ≤ 0.01. Unpaired *t*-test was used to compare between two groups. (**D**) Representative immunohistochemistry images of aortic sections stained with endothelial cell marker CD-31 (anti-CD31), and also for 4-HNE (anti-4-HNE). DAPI (blue) staining is used to detect the nucleus. Arrowheads are in the luminal side pointing towards the respective staining. Asterisk represents the smooth muscle area. Results are representative pictures of 5 (sham) and 5 (chronic anemic) independent experiments.

To investigate whether CA is associated with enhanced endothelial activation, we assessed plasma ICAM-1 and VCAM-1 level. The results showed that both ICAM-1 and VCAM-1 levels were increased in CA mice compared to sham mice (Figure 5A). Strikingly, we also observed increased plasma ICAM-1 and VCAM-1 levels in chronic coronary syndrome (CCS) patients with anemia compared to non-anemic patients (Figure 5B). Additionally, assessment of the systemic inflammatory markers demonstrated that IL6 and IL17A were progressively increased with duration of anemia (Supplementary Figure S2) demonstrating systemic inflammation in CA mice. To assess whether anemia leads to increased ROS generation in the aorta, we quantified generation of ROS-derived tyrosine adducts in the aortic lysates from anemic mice and control mice. Indeed, CA mice showed an enhanced 3-Nitrotyrosine levels in the aorta (Figure 5C). These results demonstrate that anemia results in the activation of endothelium and increased ROS production. Immunohistochemistry assessment of 4-hydroxynonenal (product of lipid peroxidation) in the aorta revealed an enhanced ROS activity in the CA mice (Figure 5D).

### ROS scavenging or MPO inhibition improves the endothelial dysfunction in chronic anemia

**Figure 6:**
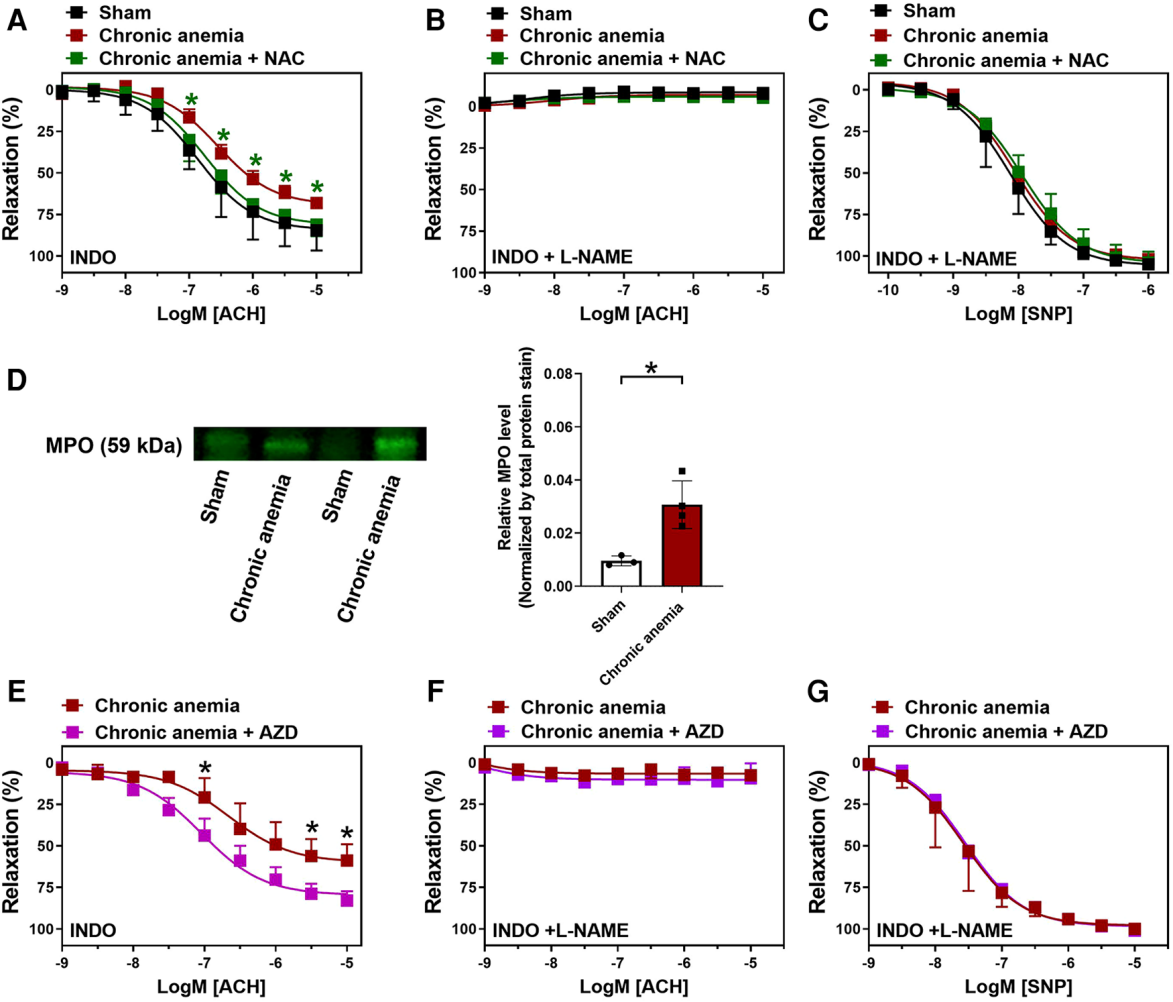
N-Acetyl cysteine (NAC) treatment or MPO inhibition improves the endothelium-dependent relaxation responses in chronic anemic mice. Aortic rings were isolated from sham (black circles), and chronic anemic mice with (green squares) and without (dark red squares) NAC supplementation. Aortic segments were pre-contracted with phenylephrine (10 µM) and relaxation responses to acetylcholine (1 nM–10 µM) were measured using an organ bath. (**A**) Relaxation in the presence of indomethacin (10 µM, COX inhibitor). (**B**) Relaxation in the presence of indomethacin and L-NAME (100 µM, NOS inhibitor). (**C**) Relaxation responses to sodium nitroprusside (SNP, 10 nM-10 µM) in the presence of indomethacin and L-NAME. (**D**) Protein level expression of MPO in the aortic lysates isolated from sham (white bar), and chronic anemic mice (red bar). In a different experimental setup (**E–G**), aortic rings from chronic anemic mice were incubated in the presence (pink squares) or absence (dark red squares) of myeloperoxidase inhibitor (AZD5904, 10 µM) and the relaxation responses were assessed to acetylcholine (**E,F**) and SNP. (**G**) All values are mean values ± SEM (*n* = 7–8 per group). *, *p* ≤ 0.05. CRCs were analysed by Two-Way ANOVA and Bonferroni's post-hoc test to compare treated (NAC or AZD) anemic group of mice with non-treated anemic mice. To compare MPO expression (**D**) Mann–Whitney test was used.

We assessed whether the ROS scavenger (NAC) improves the endothelial function and thus ED in CA mice. Aortic rings isolated from CA mice which were supplemented with NAC showed significantly improved endothelium-dependent relaxation responses (E_max_ 81 ± 3%) compared to untreated CA mice (E_max_ 68 ± 4%) (Figure 6A; Table 1). These relaxation responses are entirely attributed to NO (Figure 6B). The endothelium-independent relaxation to SNP was preserved in both groups (Figure 6C). A recent study showed that MPO expression is enhanced in ED as a consequence of vascular inflammation (15). Interestingly, the aortic lysates from CA mice also showed an increased expression of MPO compared to sham mice (Figure 6D, Supplementary Figure S5). MPO expression was also enhanced in the aortic endothelium of chronic anemic mice (Supplementary Figure S3). In addition, aortic rings treated with MPO inhibitor (AZD5904, 10 µM) showed significantly improved the relaxation responses in CA mice (Figures 6E–G; Table 1). These results conclude the potential role of ROS in anemia-associated ED.

### RBCs from anemic patients induce endothelial dysfunction

**Figure 7:**
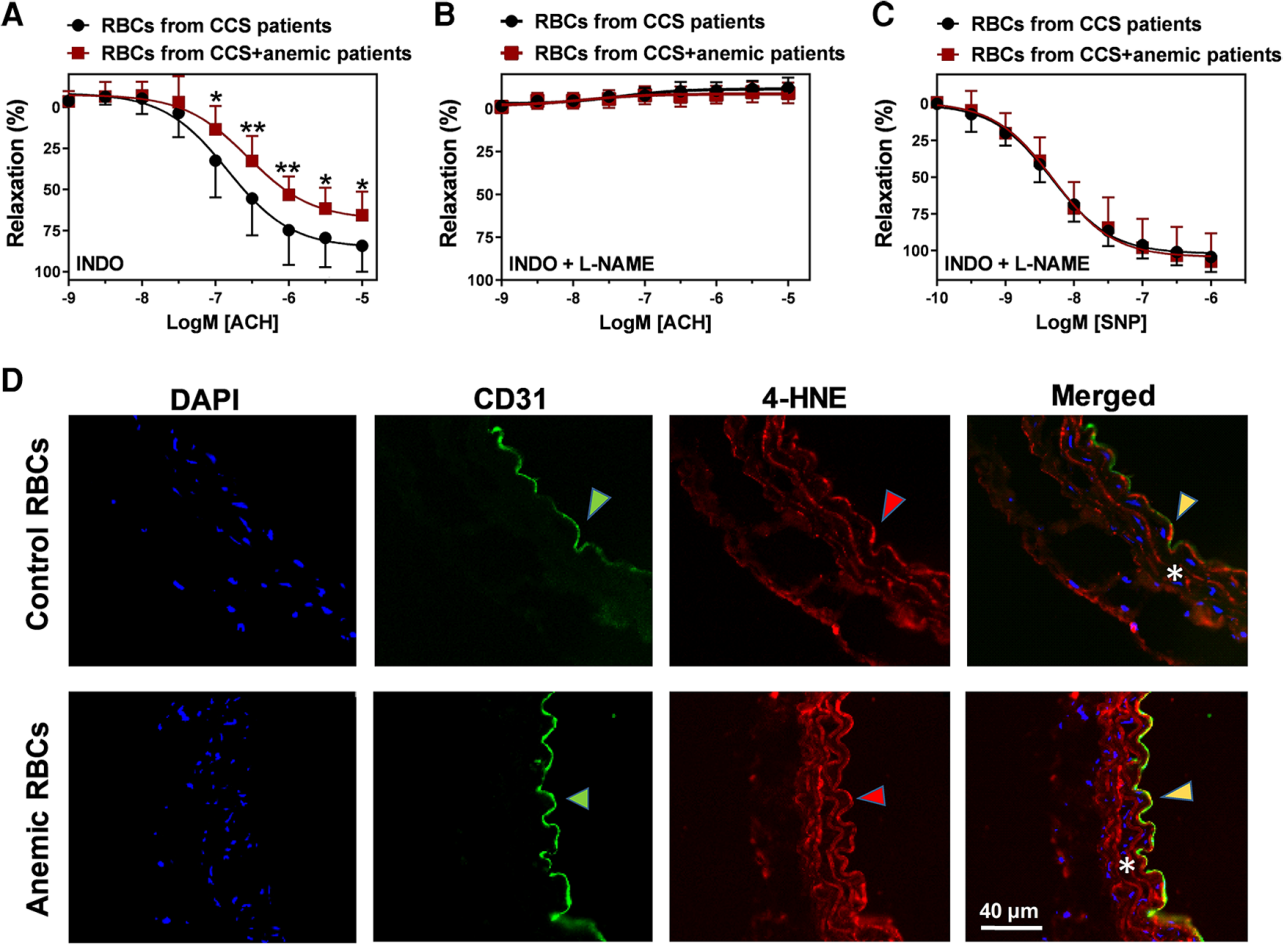
RBCs from anemic patients induce endothelial dysfunction in murine aortic rings. RBCs were isolated from chronic coronary syndrome (CCS) patients with anemia (red squares) and without anemia (black circles). Haematocrit (40%) was prepared in a KRB buffer. Aortic rings were incubated with haematocrit for 6 h at 37°C, mounted in a wire-myograph and pre-contracted with phenylephrine (10 µM). (**A**) Relaxation responses to acetylcholine (1 nM–10 µM) in the presence of indomethacin (10 µM, COX inhibitor). (**B**) Relaxation responses to acetylcholine in the presence of L-NAME (100 µM, NOS inhibitor) and indomethacin. (**C**) Endothelium-independent relaxation responses to (SNP, 10 nM-10 µM, NO donor). Values are shown as means ± SEM. *, *p* ≤ 0.05; **, *p* ≤ 0.01. CRCs were analysed by Two-Way ANOVA and Bonferroni's post-hoc test. Control group (stable CCS), *n* = 10; anemic group (CCS + Anemia), *n* = 12. (**D**) Thoracic aortas isolated from WT mice and incubated for 6 h at 37°C with RBCs from anemic or control patients. Representative immunohistochemistry images of aortic sections stained with endothelial cell marker CD-31 (anti-CD31), and 4-HNE (anti- 4-HNE). DAPI (blue) staining is used to detect the nucleus. Arrowheads are in the luminal side pointing towards the respective staining. Asterisk represents the smooth muscle area. Results are representative pictures of 5 (sham) and 5 (chronic anemic) independent experiments.

We further evaluated whether the dysfunctional RBCs impair the endothelial-dependent relaxation responses in anemia. Aortic rings from WT mice were incubated with RBCs (Hct 40%) isolated from CCS patients with and without anemia for 6 h at 37°C. The respective patients’ characteristics are summarized in Supplementary Table S1. In a separate set of experiments, aortic rings from WT mice were incubated with RBCs (Hct 40%) from anemic mice and sham mice. Evaluation of vascular function using wire-myograph demonstrated that aortic segments incubated with RBCs from anemic CCS patients show a reduced ACH-induced relaxation response (E_max_ 65 ± 5%; Figure 7A) compared to aortic segments incubated with RBCs of non-anemic CCS patients (E_max_ 82 ± 5%; Figure 7A). Inhibition of NOS by L-NAME demonstrated that these relaxation responses are mediated by NO (Figure 7B). Endothelium-independent relaxation responses to SNP were similar in both groups (Figure 7C). We also observed altered endothelium-dependent and independent relaxation responses in the aortic rings incubated with anemic mice aortic RBCs (Supplementary Figure S4). In addition, elevated expression of 4-HNE was observed in the aortic rings incubated with RBCs from anemic patients compared to aortic rings incubated with non-anemic patients (Figure 7D). Taken altogether, these results demonstrate that RBCs from anemic patients induce ROS production in the endothelium resulting in endothelial dysfunction due to altered NO-mediated relaxation responses.

## Discussion

In this study, we evaluated the effects of chronic anemia on ED in an established blood loss anemia mouse model with a hypothesis that CA alters endothelial function and lead to ED. The major findings of the study are that (1) anemic mice show impaired *in vivo* FMD responses, and abrogated endothelial NO-dependent relaxation responses *in vitro*, (2) the abrogated endothelial-dependent relaxation responses in CA are not attributed to enhanced arginase activity, (3) eNOS expression is preserved whereas enhanced iNOS expression is observed in vascular smooth muscle cells of CA mice, (4) CA is associated with increased endothelial activation and ROS production, (5) scavenging of ROS improved the endothelial function in CA mice, (6) RBCs from anemic patients induce ED in murine aortic rings.

We used a blood-loss CA mouse model to assess ED. In this model, we draw blood < 15% of the circulating volume/day on every third day for six weeks. We replaced the blood loss volume with 350 µl NaCl (0.9%) or saline to avoid any volume changes. This resulted in moderate anemia with increased plasma erythropoietin and soluble transferrin receptor levels. We previously demonstrated that arterial lactate concentration and the distribution of Evans blue dye remained within the normal range in this model indicating no signs of hemorrhage or rheological volume changes (19).

Decreased NO-dependent relaxation responses are a hallmark of ED (24, 25). Severe anemia in hemoglobinopathies such as sickle cell disease or β-thalassemia is associated with ED in both humans (26) and in respective mouse models (27, 28). Our moderate blood loss CA mouse model showed decreased NO-dependent relaxation responses similar to mouse models of hemoglobinopathies. However, the ED in hemoglobinopathies is majorly attributed to increased inflammation and hemolysis, which are mildly elevated in our CA model (Supplementary Figure S2). To prove that RBCs from anemic patients induce ED, we performed co-incubation experiments by incubating RBCs from anemic patients with mouse thoracic aortic rings. We used an adapted incubation protocol as described previously (22). In line with our hypothesis, the aortic rings incubated with RBCs of anemic patients showed reduced NO-dependent relaxation responses, which indicate that RBCs induce ED. The exact signalling mechanism of how anemic RBCs induce ED is not known. We believe that the altered secretome of RBCs might contribute to this. In addition, a recent study showed the possible role of RBC-derived extracellular vesicles in anemia-associated ED (29). We recently showed that acute (3 days) blood loss anemia is associated with a transient increase in FMD responses because of compensatory increases in cardiac output associated with increased shear stress at the endothelial surface (19). Here we demonstrate unequivocally that the acute response to anemia is compromised by progressive decrease in FMD responses in CA mice correlating with the duration of anemia. In addition, inflammatory markers such as IL6 and IL-17A were unchanged in a subacute anemic mouse model, however, these markers are significantly increased in chronic anemia. Based on our previous publication (19) and the present research work, we summarized the vascular phenotypic differences between acute and chronic anemic mouse models in Supplementary Figure S6. The differences between the two models might be explained by acute and chronic adaptation of the vascular system to anemia. We believe that in acute anemia the vascular system undergoes compensatory changes to improve the endothelial function, especially at peripheral level. However, these compensatory effects are compromised over the time.

Enhanced arginase expression/activity was known to be one of the detrimental mechanisms for ED (30). In our study, endothelial-specific deletion of arginase 1 did not improve the relaxation responses in CA. It has been shown that arginase 2 is also highly expressed in endothelium and contributes to ED in different disease states (8). We used a classic pharmacological approach to inhibit both arginases to further evaluate the possible role of arginase 2 in CA. The aortic rings from anemic mice which were treated with Nor-NOHA did not show any improvement in the relaxation responses. These results largely exclude the potential roles of arginases in ED associated with CA.

We determined whether the reduced relaxation responses in CA are also associated with decreased eNOS expression in endothelial cells. However, eNOS expression at mRNA and protein levels was preserved in CA mice. These results are also in line with previous studies on β-thalassemia anemic mouse model (27). Of note, like in other pathologies (31, 32), the inflammatory iNOS is significantly increased in the vascular smooth muscle cells (VSMC) of CA mice. The enhanced expression of iNOS in inflammatory disease state such as sepsis is known to be detrimental. Additionally, our preliminary findings showed that interleukin IL-6 and interleukin IL-17A levels are significantly elevated in CA mice which might contribute to the enhanced iNOS expression in VSMC. The mechanistic aspects of IL-6 and IL-17A -mediated iNOS is beyond the current research focus but will be studied in the future. The increased circulatory VCAM-1 and ICAM-1 levels in CA mice further underline these findings implicating the role of anemia in inflammation and associated changes in the endothelium.

It is very well known that inflammation and oxidative stress concur with each other in vascular diseases (33). Taking this into consideration, we assessed 3-Nitrotyrosine levels in CA mice. In line with the recent study on iron deficiency mouse model (34), we also observed increased 3-Nitrotyrosine levels in the aortic lysates of CA mice indicating enhanced oxidative stress. NAC is known to be a potential ROS scavenger, which has beneficial effects on endothelial function in various CVDs (35). In our CA mouse model, supplementation of NAC completely reversed the impaired NO-dependent relaxation responses, concluding the potential role of ROS in mediating oxidative stress in CA. We also believe that the preserved eNOS expression and impaired NO-dependent relaxation responses in CA are associated with oxidative stress-mediated eNOS-uncoupling, which was also reported in other disease models (27, 36). Noteworthy, we cannot rule out some limitations of this study. Herein, we mainly focused on the effects of anemia on the endothelium. However, the exact mechanism how anemic RBCs induces ED requires further investigation. Our *in vitro* studies showed that MPO inhibition with AZD5904 improves endothelial function in CA mice aortic rings. However, further *in vivo* studies are needed to evaluate the beneficial effects of this MPO inhibitor in improving ED.

## Conclusions

Our data suggest that chronic anemia is associated with decreased NO production due to increased inflammation and ROS production. Additionally, we also showed that RBCs from anemic patients attenuates the endothelial relaxation responses concluding the potential role of anemic RBCs in ED. We demonstrated that increased ROS production in the aortic endothelium of anemic mice, and treating the anemic mice with ROS scavenger (NAC) improved the relaxation responses in CA. In addition, MPO expression in endothelium is elevated in CA mice, and the MPO inhibitor AZD5904 also improved the relaxation responses in CA mice. Taken together, NAC supplementation and/or MPO inhibition improves endothelial function in anemia.

## Data Availability

The original contributions presented in the study are included in the article/**Supplementary Material**, further inquiries can be directed to the corresponding authors.
