# Supplementary material for: Chronic anemia is associated with systemic endothelial dysfunction
Source: Front Cardiovasc Med. 2023 May 10;10:1099069. doi: 10.3389/fcvm.2023.1099069 (PMC10205985; doi:10.3389/fcvm.2023.1099069)
Supplement: Supplementary file 1 [file Presentation1.pptx]

## Slide 1
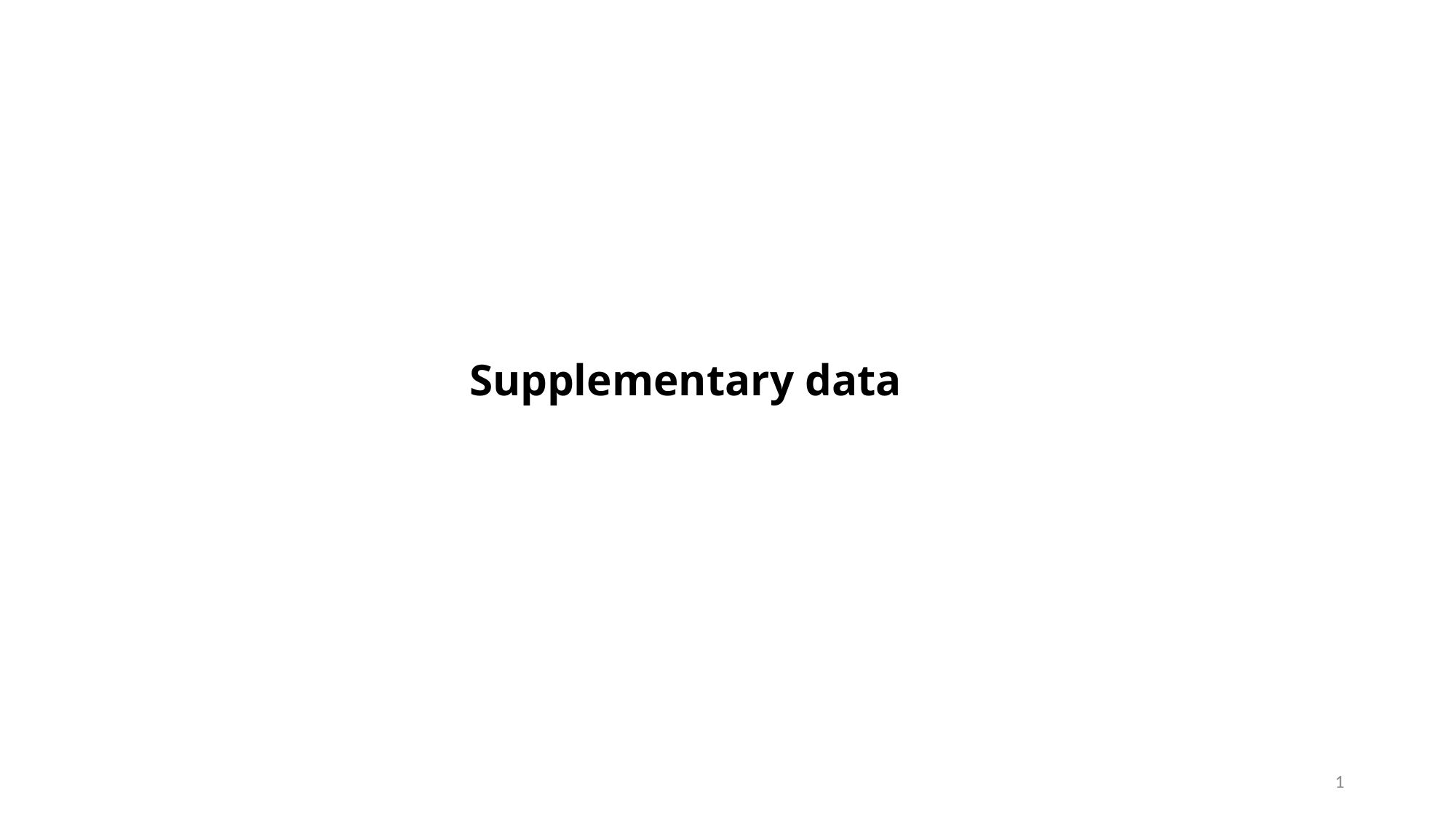

# Supplementary data
1

## Slide 2
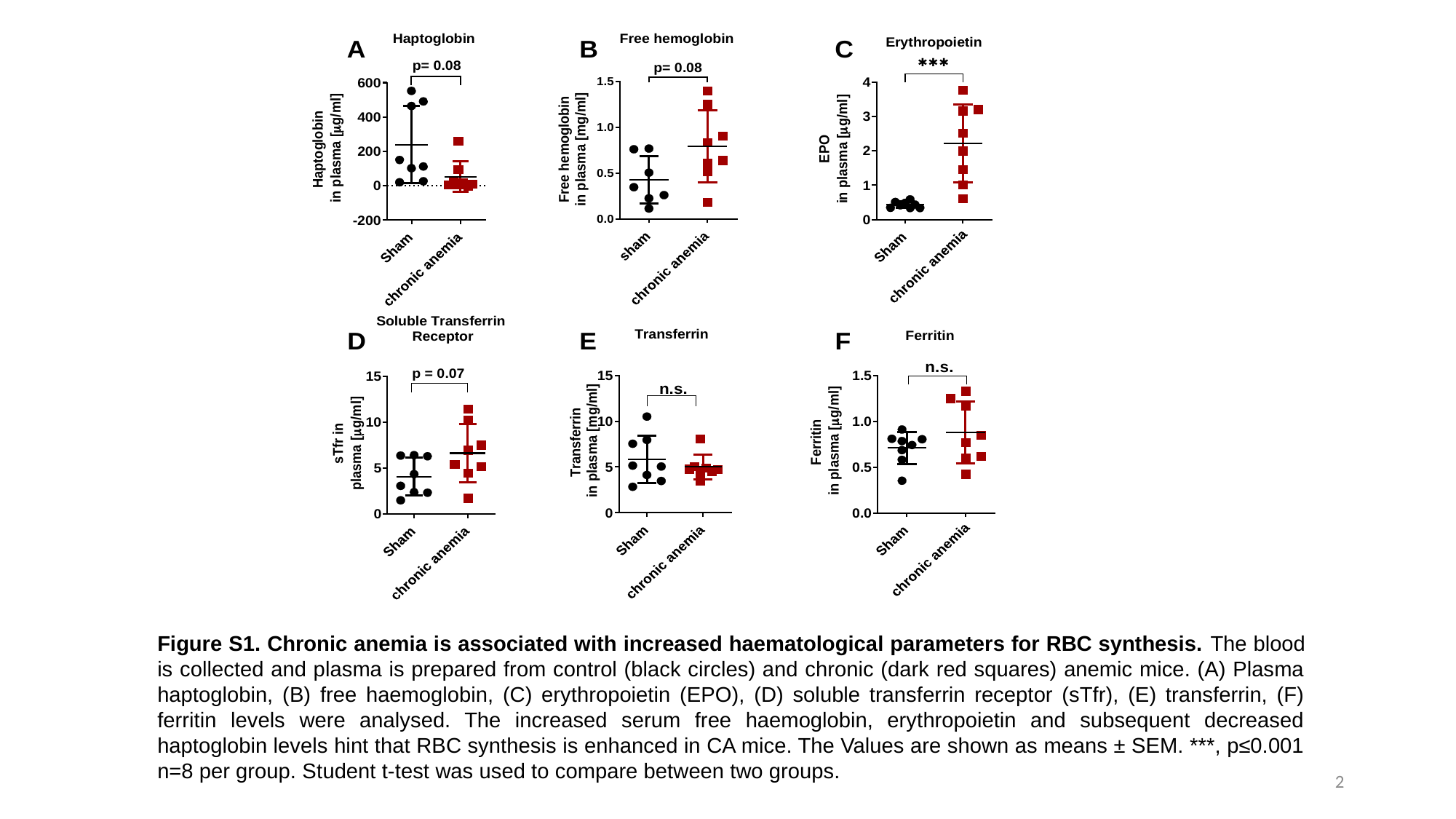

Figure S1. Chronic anemia is associated with increased haematological parameters for RBC synthesis. The blood is collected and plasma is prepared from control (black circles) and chronic (dark red squares) anemic mice. (A) Plasma haptoglobin, (B) free haemoglobin, (C) erythropoietin (EPO), (D) soluble transferrin receptor (sTfr), (E) transferrin, (F) ferritin levels were analysed. The increased serum free haemoglobin, erythropoietin and subsequent decreased haptoglobin levels hint that RBC synthesis is enhanced in CA mice. The Values are shown as means ± SEM. ***, p≤0.001 n=8 per group. Student t-test was used to compare between two groups.
2

## Slide 3
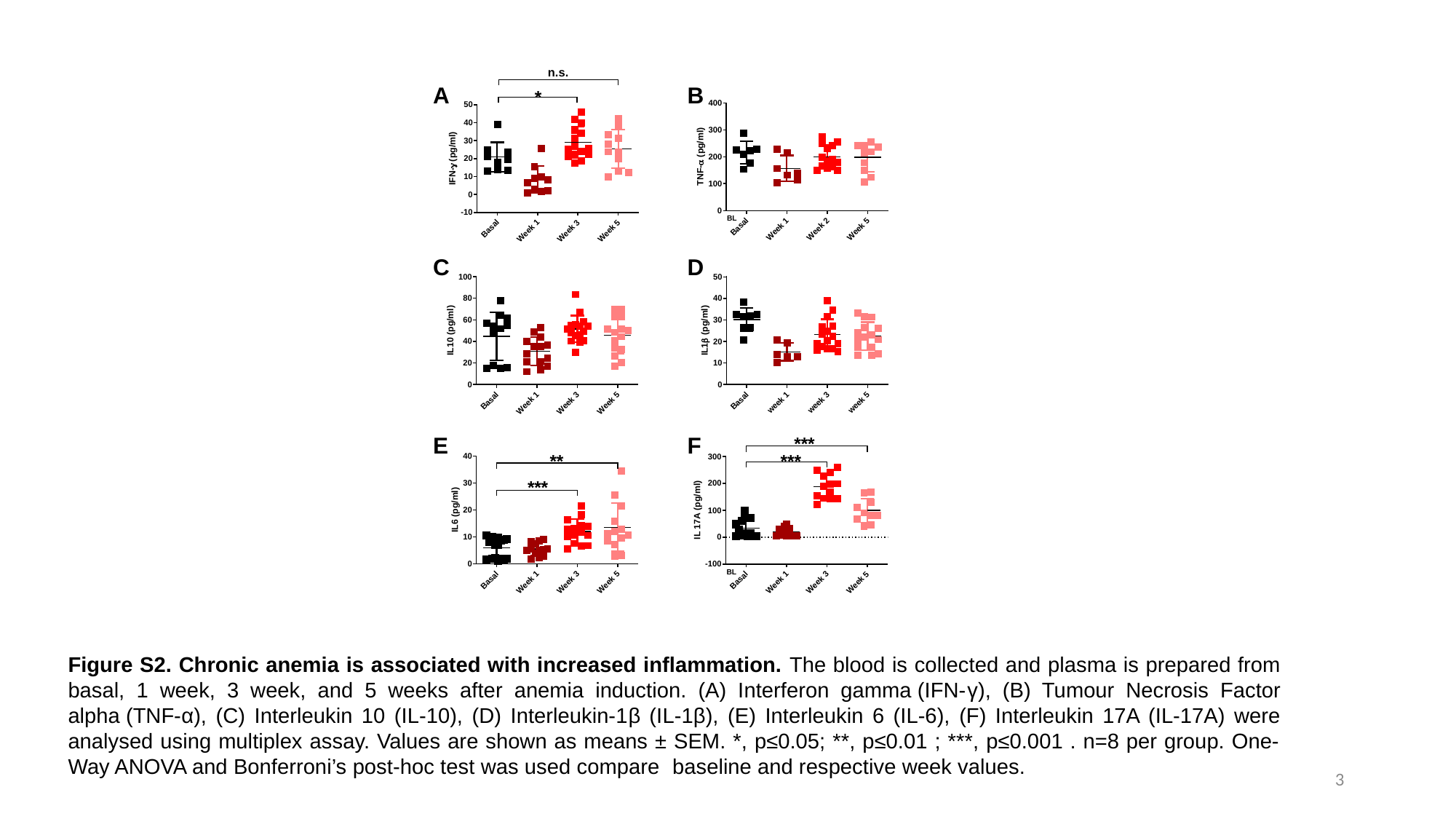

Figure S2. Chronic anemia is associated with increased inflammation. The blood is collected and plasma is prepared from basal, 1 week, 3 week, and 5 weeks after anemia induction. (A) Interferon gamma (IFN-γ), (B) Tumour Necrosis Factor alpha (TNF-α), (C) Interleukin 10 (IL-10), (D) Interleukin-1β (IL-1β), (E) Interleukin 6 (IL-6), (F) Interleukin 17A (IL-17A) were analysed using multiplex assay. Values are shown as means ± SEM. *, p≤0.05; **, p≤0.01 ; ***, p≤0.001 . n=8 per group. One-Way ANOVA and Bonferroni’s post-hoc test was used compare baseline and respective week values.
3

## Slide 4
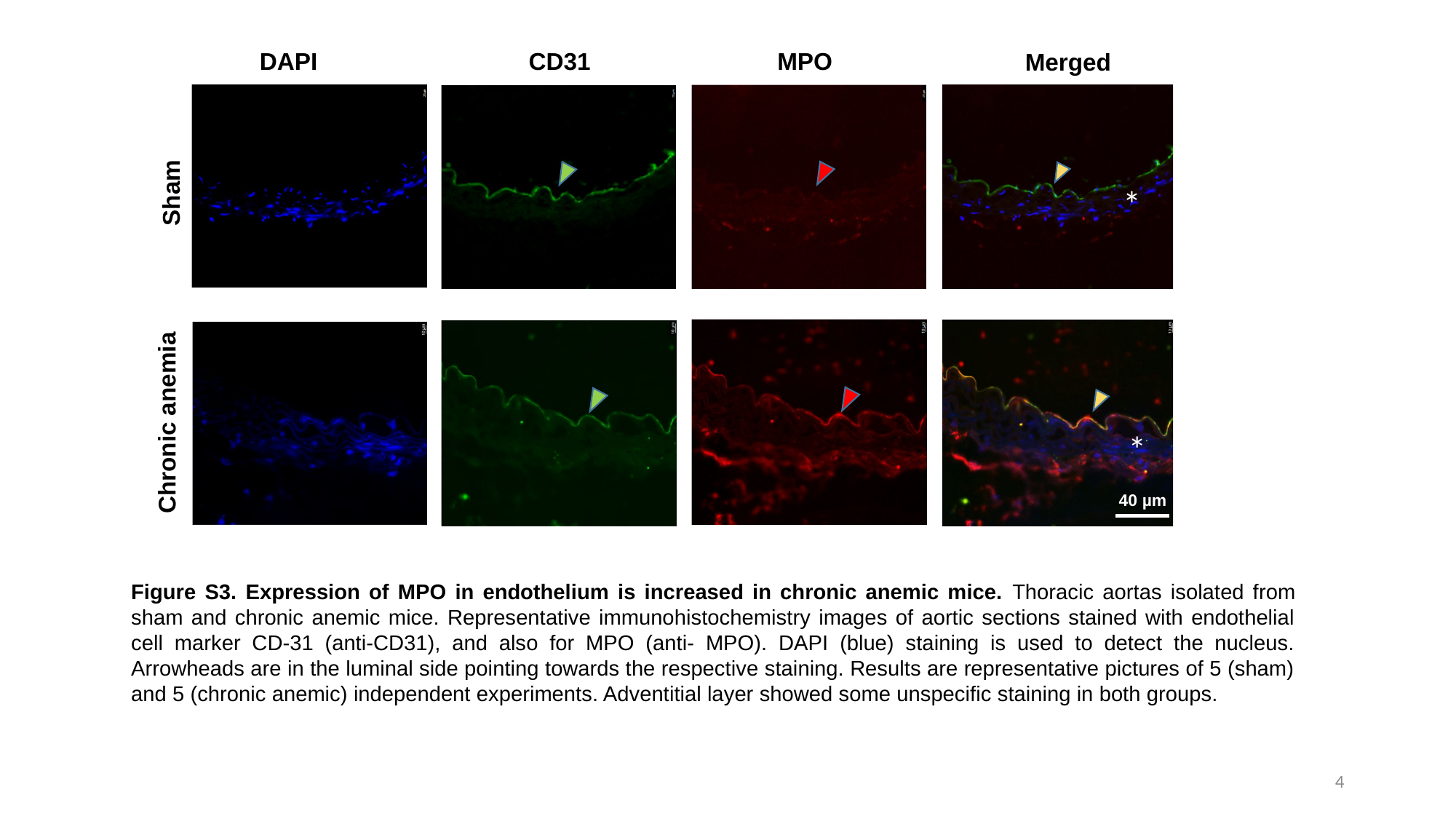

CD31
MPO
DAPI
Merged
*
Sham
*
40 µm
Chronic anemia
Figure S3. Expression of MPO in endothelium is increased in chronic anemic mice. Thoracic aortas isolated from sham and chronic anemic mice. Representative immunohistochemistry images of aortic sections stained with endothelial cell marker CD-31 (anti-CD31), and also for MPO (anti- MPO). DAPI (blue) staining is used to detect the nucleus. Arrowheads are in the luminal side pointing towards the respective staining. Results are representative pictures of 5 (sham) and 5 (chronic anemic) independent experiments. Adventitial layer showed some unspecific staining in both groups.
4

## Slide 5
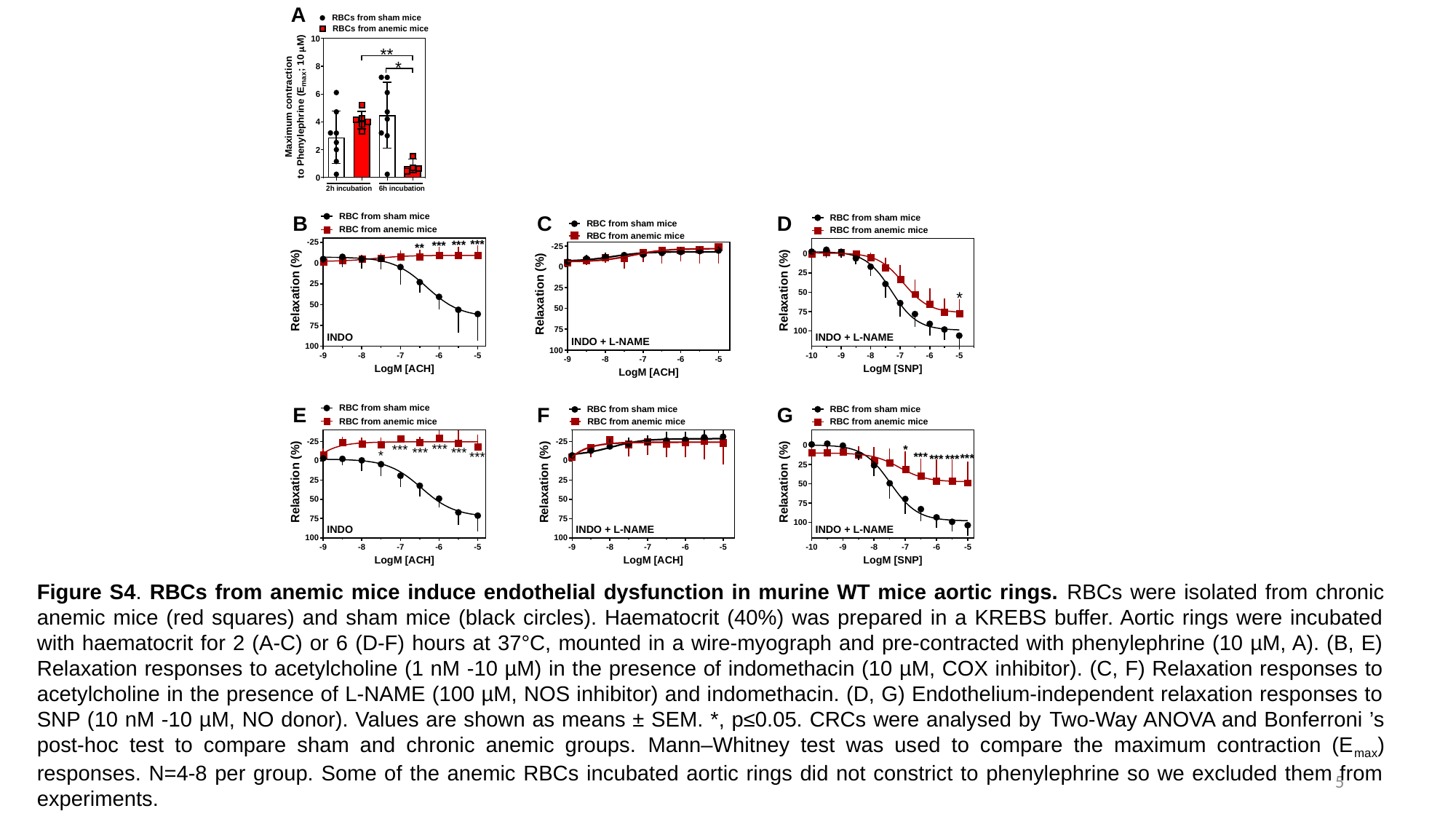

Figure S4. RBCs from anemic mice induce endothelial dysfunction in murine WT mice aortic rings. RBCs were isolated from chronic anemic mice (red squares) and sham mice (black circles). Haematocrit (40%) was prepared in a KREBS buffer. Aortic rings were incubated with haematocrit for 2 (A-C) or 6 (D-F) hours at 37°C, mounted in a wire-myograph and pre-contracted with phenylephrine (10 µM, A). (B, E) Relaxation responses to acetylcholine (1 nM -10 µM) in the presence of indomethacin (10 µM, COX inhibitor). (C, F) Relaxation responses to acetylcholine in the presence of L-NAME (100 µM, NOS inhibitor) and indomethacin. (D, G) Endothelium-independent relaxation responses to SNP (10 nM -10 µM, NO donor). Values are shown as means ± SEM. *, p≤0.05. CRCs were analysed by Two-Way ANOVA and Bonferroni ’s post-hoc test to compare sham and chronic anemic groups. Mann–Whitney test was used to compare the maximum contraction (Emax) responses. N=4-8 per group. Some of the anemic RBCs incubated aortic rings did not constrict to phenylephrine so we excluded them from experiments.
5

## Slide 6
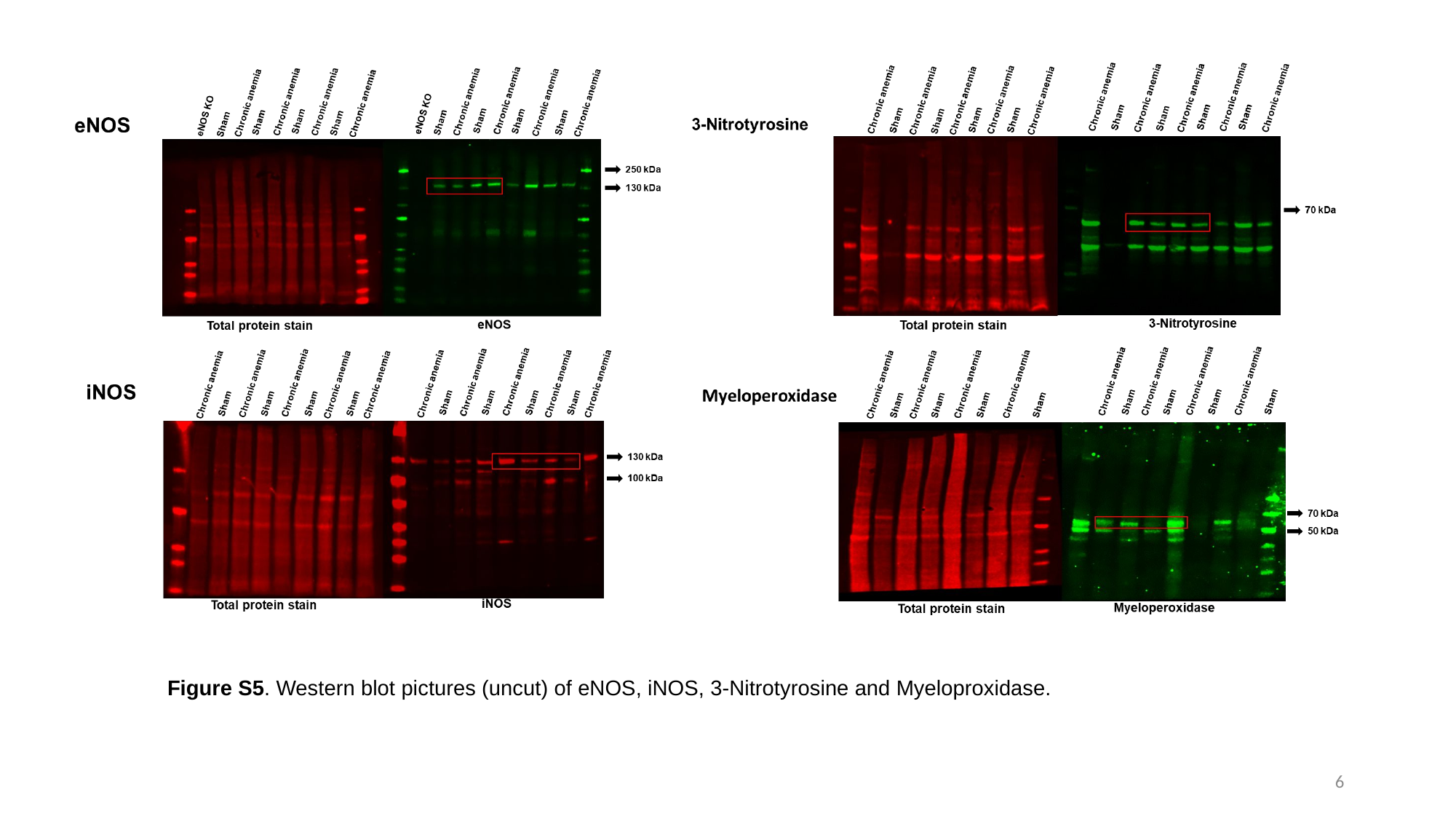

Figure S5. Western blot pictures (uncut) of eNOS, iNOS, 3-Nitrotyrosine and Myeloproxidase.
6

## Slide 7
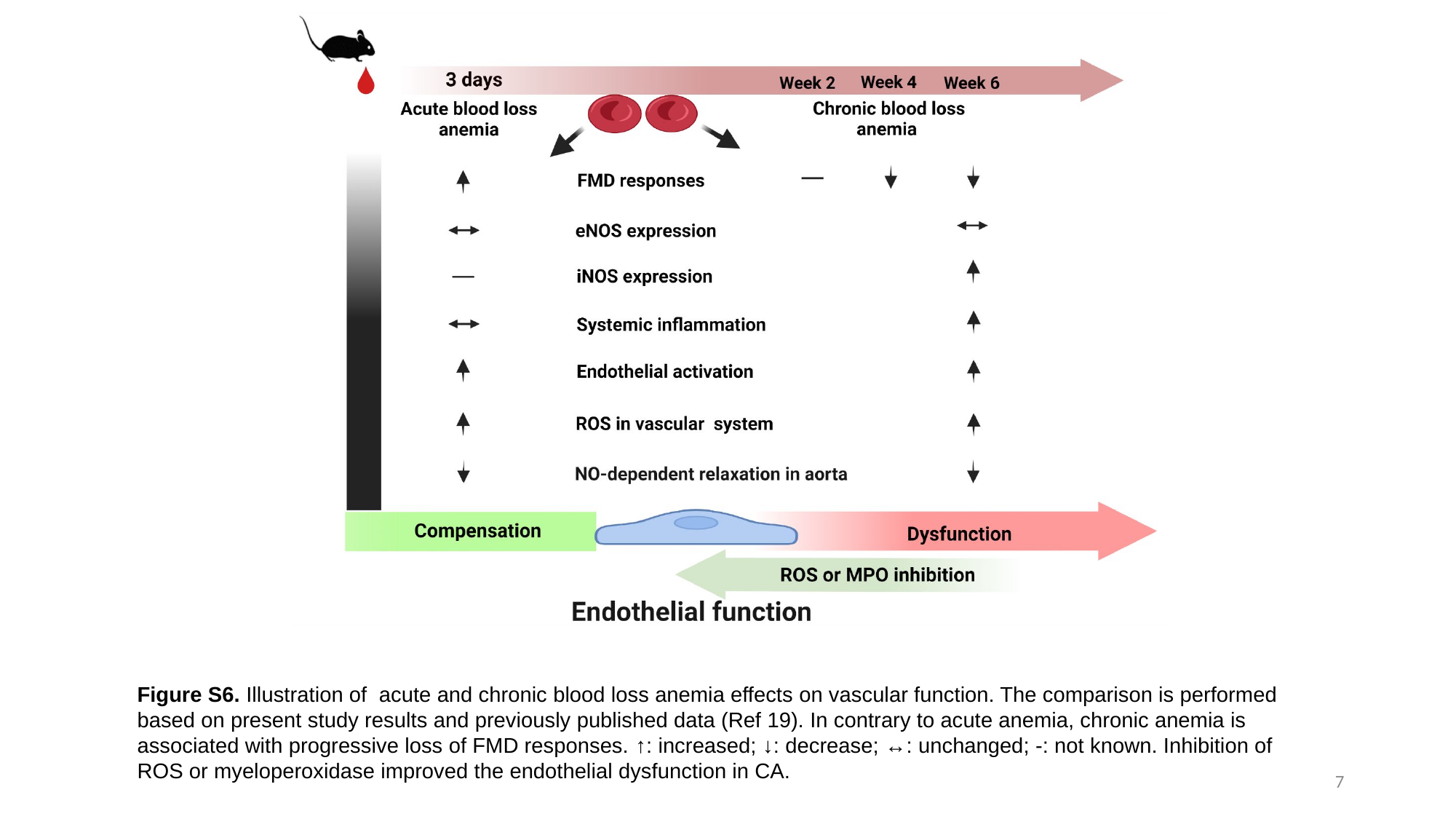

Figure S6. Illustration of acute and chronic blood loss anemia effects on vascular function. The comparison is performed based on present study results and previously published data (Ref 19). In contrary to acute anemia, chronic anemia is associated with progressive loss of FMD responses. ↑: increased; ↓: decrease; ↔: unchanged; -: not known. Inhibition of ROS or myeloperoxidase improved the endothelial dysfunction in CA.
7

## Slide 8
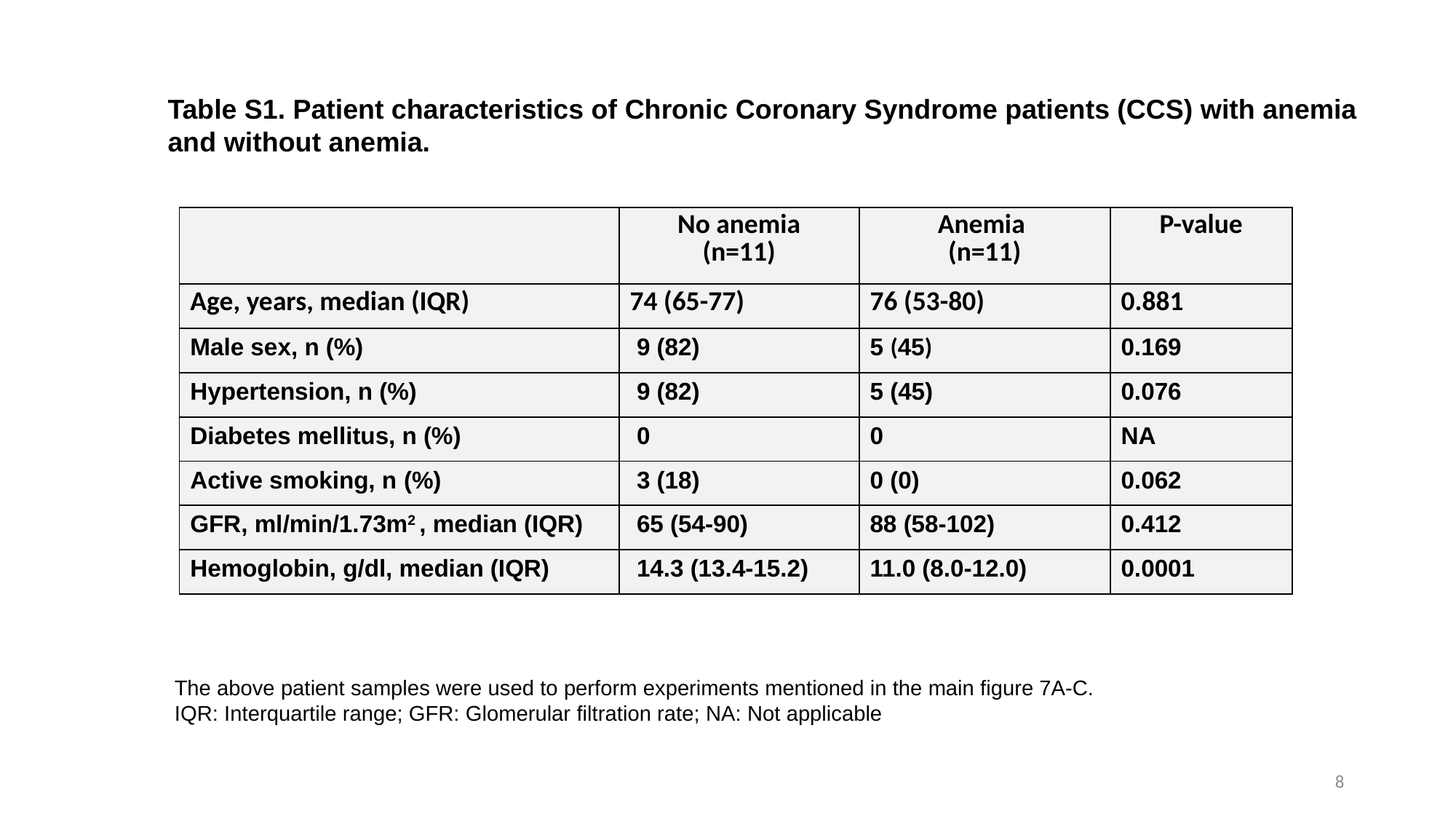

Table S1. Patient characteristics of Chronic Coronary Syndrome patients (CCS) with anemia and without anemia.
| | No anemia (n=11) | Anemia (n=11) | P-value |
| --- | --- | --- | --- |
| Age, years, median (IQR) | 74 (65-77) | 76 (53-80) | 0.881 |
| Male sex, n (%) | 9 (82) | 5 (45) | 0.169 |
| Hypertension, n (%) | 9 (82) | 5 (45) | 0.076 |
| Diabetes mellitus, n (%) | 0 | 0 | NA |
| Active smoking, n (%) | 3 (18) | 0 (0) | 0.062 |
| GFR, ml/min/1.73m2 , median (IQR) | 65 (54-90) | 88 (58-102) | 0.412 |
| Hemoglobin, g/dl, median (IQR) | 14.3 (13.4-15.2) | 11.0 (8.0-12.0) | 0.0001 |
The above patient samples were used to perform experiments mentioned in the main figure 7A-C.
IQR: Interquartile range; GFR: Glomerular filtration rate; NA: Not applicable
8

## Slide 9
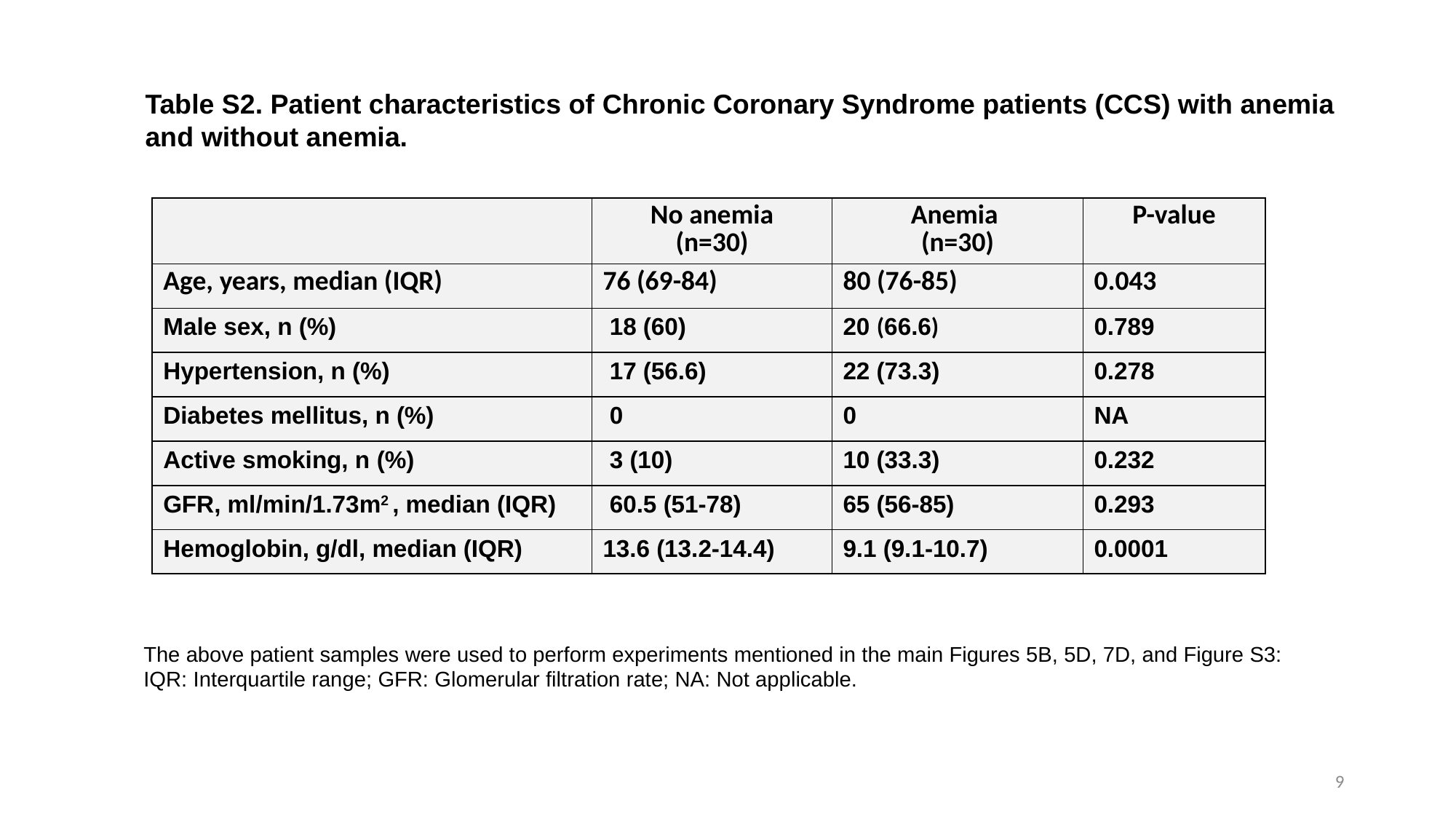

Table S2. Patient characteristics of Chronic Coronary Syndrome patients (CCS) with anemia and without anemia.
| | No anemia (n=30) | Anemia (n=30) | P-value |
| --- | --- | --- | --- |
| Age, years, median (IQR) | 76 (69-84) | 80 (76-85) | 0.043 |
| Male sex, n (%) | 18 (60) | 20 (66.6) | 0.789 |
| Hypertension, n (%) | 17 (56.6) | 22 (73.3) | 0.278 |
| Diabetes mellitus, n (%) | 0 | 0 | NA |
| Active smoking, n (%) | 3 (10) | 10 (33.3) | 0.232 |
| GFR, ml/min/1.73m2 , median (IQR) | 60.5 (51-78) | 65 (56-85) | 0.293 |
| Hemoglobin, g/dl, median (IQR) | 13.6 (13.2-14.4) | 9.1 (9.1-10.7) | 0.0001 |
The above patient samples were used to perform experiments mentioned in the main Figures 5B, 5D, 7D, and Figure S3:
IQR: Interquartile range; GFR: Glomerular filtration rate; NA: Not applicable.
9
